# Supplementary material for: Synaptic gene expression changes in frontotemporal dementia due to the MAPT 10+16 mutation
Source: medRxiv. 2024 Apr 12:2024.04.09.24305501. Preprint. [Version 1] doi: 10.1101/2024.04.09.24305501 (PMC11030522; doi:10.1101/2024.04.09.24305501)
Supplement: Supplement 1 [file NIHPP2024.04.09.24305501v1-supplement-1.pdf]

medRxiv preprint doi: https://doi.org/10.1101/2024.04.09.24305501; this version posted April 12, 2024. The copyright holder for this preprint
(which was not certified by peer review) is the author/funder, who has granted medRxiv a license to display the preprint in perpetuity.
It is made available under a CC-BY 4.0 International license .

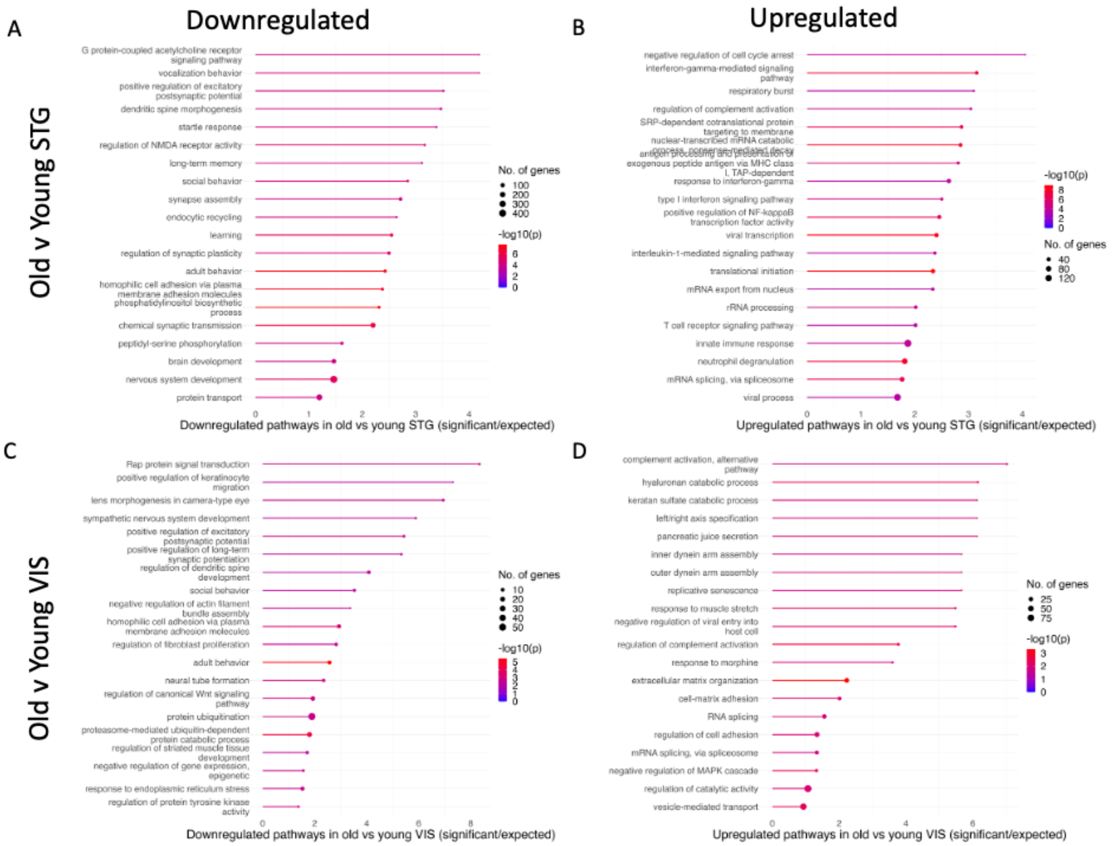

Supplementary Figure 1: When comparing older vs younger controls (cutoff 65 years of age), the top 20 down-regulated (A, C) and up-regulated (B,D) pathways in Gene Ontology analysis of RNAseq data reveal that in both STG (A, B), and visual cortex (C, D), expression of genes involved in synaptic function are decreased and genes involved in inflammation and regulation of the cell cycle are increased.
